# Tryptophan Metabolites as Mediators of Microbiota-Gut-Brain Communication: Focus on Isatin

**DOI:** 10.3389/fnbeh.2022.922274

**Published:** 2022-06-30

**Authors:** Alexei Medvedev, Olga Buneeva

**Affiliations:** Laboratory of Pharmacoproteomics, Institute of Biomedical Chemistry, Moscow, Russia

**Keywords:** tryptophan metabolites, isatin, gut-brain axis, brain, molecular targets, isatin-binding proteins, protein-protein interactions, interactome

## Abstract

Isatin (indole-2,3-dione) is an endogenous regulator, exhibiting various behavioral, biological, and pharmacological activities. Synthesis of isatin includes several crucial stages: cleavage of the tryptophan side chain and subsequent oxidation of the indole nucleus. Although these stages require concerted action of bacterial and host enzymes, there are two pathways of isatin formation: the host and bacterial pathways. Isatin acts as a neuroprotector in different experimental models of neurodegeneration. Its effects are realized *via* up- and downregulation of isatin-responsive genes and *via* interaction with numerous isatin-binding proteins identified in the brain. The effect of isatin on protein-protein interactions in the brain may be important for realization of weak inhibition of multiple receptor targets.

## Introduction

It becomes increasingly clear that gut microbiota has a significant impact on brain functioning (Cryan et al., 2019). Convincing evidence now exists that the human gut microbiome is involved in numerous neurological processes, neurodevelopment, behavior and aging as well as neurodegenerative diseases (Sampson and Mazmanian, 2015; Kennedy et al., 2017). In stress-sensitive germ-free F344 rats, the lack of gut microbiota exacerbated neuroendocrine and behavioral responses to acute stress (Crumeyrolle-Arias et al., 2014).

Among many metabolites produced by microorganisms colonized the gastrointestinal tract, tryptophan metabolites are of special interest. Tryptophan (Trp) is an essential proteinogenic amino acid, primarily derived from dietary sources. Besides its role as a building block for protein synthesis, Trp enters various metabolic pathways, in which its metabolites, generated by host and microbial cells, act as interspecies and interkingdom signaling molecules (Lee et al., 2015). Host cells metabolize dietary Trp into tryptamine, 5-hydroxytryptophan, 5-hydroxytryptamine (5-HT, serotonin), kynurenine (Kyn), and their downstream derivatives (Palego et al., 2016; Grifka-Walk et al., 2021). In turn, microbes recycle free Trp and generate indole-containing compounds (Lee et al., 2015; Palego et al., 2016; Grifka-Walk et al., 2021). Altered tryptophan levels influence mood and behavior in humans (Young et al., 1988). Altered 5-HT transmission is associated with mood-affective disorders, autism and cognitive deficit, anorexia or bulimia nervosa, and obesity (Hudson and Pope, 1996; Gingrich and Hen, 2001; Whitaker-Azmitia, 2001; Giannaccini et al., 2013; Abdul Rahim et al., 2019).

In the context of Trp-derived signaling molecules, acting as interspecies and interkingdom signaling molecules, isatin, indole-2,3-dione, is of special interest (Cryan et al., 2019). Isatin, a bacterial and host metabolite of Trp/indole (Sandler et al., 1991; Cryan et al., 2019), demonstrates a wide range of bevarioral, biological, and pharmacological activities (see for review Medvedev et al., 2018), including anxiety-like behavior in rodents. Various types of stress have a significant impact on isatin levels in the brain, serum, urine, and examined tissues. In rats exposed to immobilization/audiogenic stress, the isatin levels in the brain (heart, and serum) were 2-4-fold higher than in control animals (Igosheva et al., 2004). Cold stress of rats (for 2 h at 4°C) significantly increased (by 2-3-fold) the isatin content in the daily urine (Tozawa et al., 1998). Food deprivation for 3 days (with free access to water) caused even more pronounced (∼5-fold) increase of isatin in the daily urine (Tozawa et al., 1998). In humans, increased level of isatin in biological body fluids was found in several neurological disorders. A significant increase in urinary isatin was observed in patients with Parkinson’s disease (stages III-V), and the urinary isatin reflected severity of this disease because it tended to increase in accordance to the Hoehn and Yahr scale (Hamaue et al., 2000). Bullimia nervosa is another pathological condition accompanied by altered isatin level evaluated in cerebrospinal fluid (Brewerton et al., 1995). It still remains unknown, which route of isatin biosynthesis contributes to the altered levels of this substance in body fluids in norm and pathology. Nevertheless, it is important to incorporate recent data, obtained during identification of molecular targets of isatin in the brain, into the context of the role of isatin as a signaling molecule with multitarget action.

## A Brief Overview of Isatin Targets in the Brain

**FIGURE 1:**
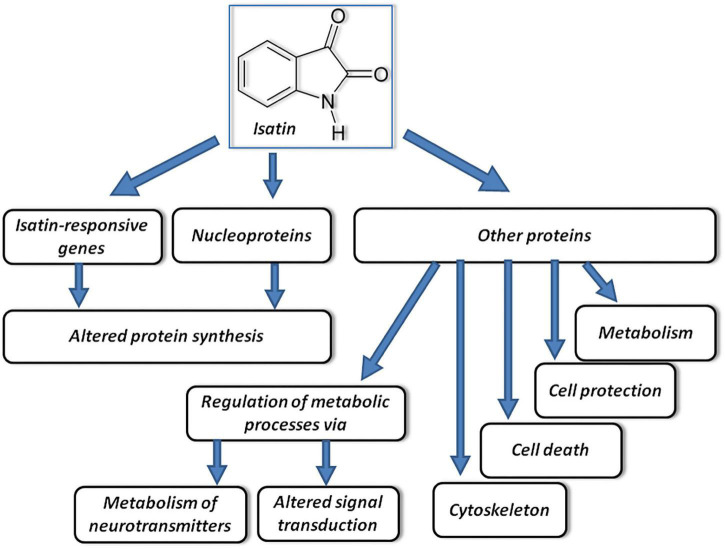
Proposed mechanisms of multitarget effects of isatin. Acting on responsive genes and certain nucleoproteins, isatin influences synthesis of proteins. Acting on other protein targets, it influences other processes schematically shown on the figure and considered in the text. Modified from Medvedev et al. (2018).

Isatin binding sites are widely distributed in the brain (Crumeyrolle-Arias et al., 2003, 2009). In the rat brain their density reduces in the following order: hypothalamus > cortex, hippocampus > cerebellum, striatum > thalamus > brain stem. Quantitative characterization of isatin binding sites in various structures of the rat brain by means of [^3^H]isatin showed that the K_*d*_ values for [^3^H]isatin binding were within the physiological range of concentrations detected in the brain and body fluids (blood, cerebrospinal fluid, and urine) (Crumeyrolle-Arias et al., 2009). In the brain, the isatin binding sites predominate in the particulate fraction (Ivanov et al., 2002). Experiments with administration of a dose of isatin, which attenuated signs of neurotoxicity of 1-methyl-4-phenyl-1,2,3,6-tetrahydropyridine (MPTP), inducing Parkinsonism in mice (Medvedev et al., 2017), revealed a multilevel action of this substance (Medvedev et al., 2020; Figure 1).

### Isatin-Responsive Genes

*In vivo*, administration of a neuroprotective dose of isatin to rats (100 mg/kg, 24 h) influenced expression of more than 850 genes, including 433 upregulated and 418 downregulated genes (Medvedev et al., 2020). However, at this time interval after the isatin-induced gene expression, changes were not accompanied by changes in their corresponding protein products. Results of transcriptome and proteome analyses of differentially expressed genes suggest the lack of concerted changes 24 h after administration of isatin. This means that changes in the transcripts of genes were not translated into corresponding changes of their protein products. In addition, unidirectional changes occurred in genes/proteins involved in opposite processes (e.g., cell proliferation and cell death). This could be associated with the phenomenon of uncoupling of transcription and translation due to the delay between transcription and translation, which occurs in the course of dynamic adaptation processes (Liu et al., 2016). Nevertheless, at 24 h after administration, the neuroprotective dose of isatin downregulated some brain-specific processes (and corresponding genes) and upregulated expression of genes involved in the stress response (Medvedev et al., 2020).

Although isatin analogs may effectively interact with DNA *in vitro* (e.g., Arshad et al., 2022), the effect of isatin on gene expression is obviously mediated by various nucleoproteins, including histones and histone modifying enzymes (e.g., histone deacetylase) (Medvedev et al., 2018; Hua et al., 2021).

### Differentially Expressed Proteins

*In vitro* studies revealed that incubation of tumor cell cultures (SH-SY5Y, MCF-7) with 50–400 μM isatin decreased expression of the studied genes and their protein products, including Bcl-2, VEGF, cyclin D1, metalloproteinases 2 and 9 (MMP-2, MMP-9), monoamine oxidase A, HIF-1α (hypoxia-inducible factor 1-alpha), CXCR4 (chemokine receptor type 4) (Hou et al., 2008; Song et al., 2013; Ma et al., 2014; Xu et al., 2016; Sun et al., 2017).

Isatin administered to mice (24 h) downregulated more than 30 brain proteins (Medvedev et al., 2020). The most pronounced changes were found in the case of calcium/calmodulin-dependent protein kinase type IV (about 11-fold), fructose-1,6-bisphosphatase 1 (more than 8-fold), serine protease inhibitor A3K (more than 4-fold), nucleolar protein 3 (almost 4-fold), neurobeachin (more than 3-fold). These proteins participate in cell signaling, regulation of cell death and proliferation. Some of these proteins exhibit moonlighting functions (i.e., they possess other activities unrelated to originally recognized activities). For example, fructose-1,6-bisphosphatase 1 (FBP1), a classical glycolytic enzyme, may block the transcriptional activity of the hypoxia-inducible factor (HIF-1α) and prevent activation of the RAS/RAF/MEK/ERK pathway (see for review Snaebjornsson and Schulze, 2018).

### Isatin-Binding Proteins

Isatin interacts with numerous isatin-binding proteins, which have been identified during affinity-based proteomic profiling of mouse and rat brain tissue lysates (Buneeva et al., 2010, 2012, 2019; Medvedev et al., 2020). Functionally they fall into the following groups: (I) Energy generation and carbohydrate metabolism; (II) Cytoskeleton formation and exocytosis/trafficking; (III) Regulation of gene expression, cell division and differentiation; (IV) Signal transduction and regulation of enzyme activity; (V) Antioxidant and protective proteins/enzymes; and (VI) Metabolism of amino acids and other nitrogenous compounds. It should be noted that the mouse and rat brain profiles of the brain isatin binding proteins demonstrate significant interspecies differences.

These interspecies differences may contribute to different sensitivity of rats and mice to prolonged immobilization stress (Armario and Castellanos, 1984) or action of chemical toxins (Cunningham, 2002). Mice are sensitive to the dopaminergic neurotoxin MPTP, whereas rats are relatively resistant to this neurotoxin (Bové and Perier, 2012). In the context of sensitivity of model animals to isatin, poor coincidence of proteins from the group of proteins/enzymes involved in cell signaling (and possibly some others) may explain known differences in responsiveness of rats and mice to isatin administration. In contrast to Bhattacharya et al. (1991), Bhattacharya and Acharya (1993), reporting anxiogenic activity of low doses of isatin in the open-field and elevated plus*-*maze tests in albino mice, Abel (1995) did not observe such effect of isatin in the open-field test in rats.

Besides species-specific isatin-binding proteins, there is a representative group of brain isatin-binding proteins common for mice and rats; it includes moonlighting proteins, which exhibit some non-canonical functions in addition to their classical activities (Medvedev et al., 2020).

Brain isatin binding proteins demonstrate differential response to administered isatin (Buneeva et al., 2019; Medvedev et al., 2020). In this regard, there are several pools of isatin binding proteins in the brain. Control specific isatin binding proteins are characteristic of intact animals and *in vitro* they bind to the immobilized isatin analog. Since isatin administration decreases/eliminates their ability to bind the immobilized isatin analog *in vitro*, it appears that they represent direct targets of isatin. This well fits to the mechanism of homologous competition, when specific binding of [^3^H]isatin to various brain structures *in vitro* decreased in the presence of unlabeled isatin (Crumeyrolle-Arias et al., 2003, 2009).

Existence of isatin-binding proteins specific of isatin-treated animals may be associated with several possible mechanisms including isatin-induced changes in protein-protein interactions as well as isatin-induced formation of new binding sites. However, regardless of particular mechanism(s) it should be noted that these isatin-binding proteins have particular cellular localization (Medvedev et al., 2020). In contrast to control-specific isatin-binding proteins, which were not linked (assigned) to the particular cell compartments, the brain isatin-binding proteins specific of the isatin-treated animals could be well characterized by targeted localization related to neuronal synapses (Medvedev et al., 2020).

Functionally, control-specific isatin-binding proteins mainly exist as independent molecules unrelated to each other: the majority of these isatin-binding proteins demonstrate rather poor functional interactions thus suggesting their involvement in distinct, unrelated processes (Medvedev et al., 2020).

In the case of brain isatin-binding proteins specific of isatin-treated mice, most proteins formed a deeply integrated protein network (Medvedev et al., 2020). In this network, a member of the membrane associated guanylate kinase (MAGUK) family of proteins, DLG4 (known as synapse-associated protein 90, SAP90 and PSD-95 protein), serves as a hub linking together several clusters of proteins, involved in: chromatin modification, cytoskeleton formation/rearrangement and intracellular trafficking, metabolic processes, posttranslational modification of proteins, and signaling. This ordered protein network containing new functional protein clusters induced by isatin may be also important for realization of weak inhibition of multiple receptor targets.

### Effects of Isatin on Neurotransmitter Receptors

Isatin was tested as a potential inhibitor of many receptors *in vitro* (Glover et al., 1995; Medvedev et al., 2007, 2018). Functional importance of inhibition of particular targets *in vivo* has been demonstrated mainly in the case of monoamine oxidase B and natriuretic peptide receptors, which demonstrate the highest sensitivity to inhibition by isatin (IC_50_ within 1–10 μM). Besides these isatin targets, there is a group of receptors, which demonstrate a weak sensitivity to inhibition by the physiological concentration of isatin (10 μM). These include NMDA receptor, AMPA receptor, Dopamine D4-receptor, Muscarinic (M-2) receptor, Glycine (strychnine sensitive) receptor. Isatin inhibited this receptor binding by 20–40%. Although each effect of isatin is weak in the context of its pharmacological effect on individual targets, such weak inhibition of multiple targets may be quite efficient (synergistic). For example, a proconvulsant effect of isatin was attributed to its action on 5HT3 receptors (Bhattacharya and Chakrabarti, 1998), while direct binding assays did not reveal any effect of isatin on this receptor subtype (Glover et al., 1995). In addition, a weak inhibition of D-amino acid oxidase (DAAO) by isatin (about 20% at 20 μM; Szilágyi et al., 2018) may contribute to modulation of NMDA receptor function (Strick et al., 2011). In related research fields, this is considered as a putative molecular basis explaining broad-spectrum of biological actions of polypharmacology agent(s) (Ágoston et al., 2005; Csermely et al., 2005; Xie et al., 2011).

## Host Bacterial Cross-Talk in Isatin Biosynthesis

**FIGURE 2:**
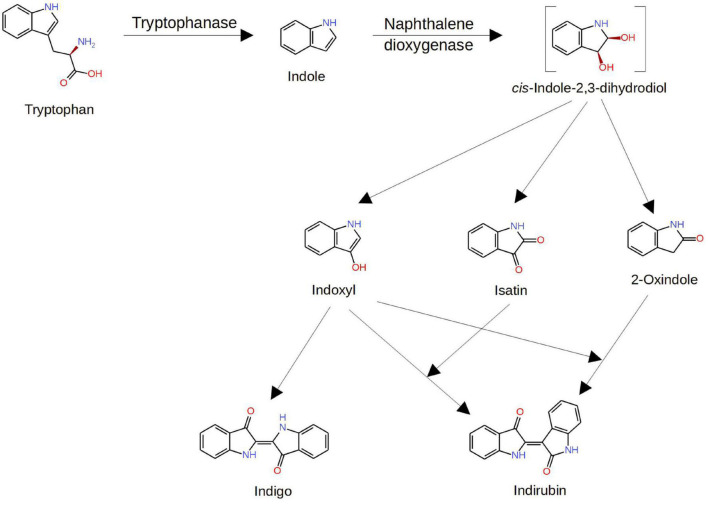
Pathways of isatin formation from tryptophan. Modified from Zhang et al. (2014). Explanations are given in the text.

Routes of metabolic conversion of tryptophan to isatin by host and microbial enzymes are more or less understood. These include cleavage of the tryptophan side chain by bacterial tryptophanase (encoded by the *TnaA* gene) and subsequent oxidation of the indole moiety, catalyzed by bacterial naphthalene dioxygenase (NDO), with formation of isatin and other downstream metabolites (Figure 2). Free indole absorbed by host cells can be metabolized by the host cytochrome P450 monooxygenase systems with formation of isatin and other products of indole oxidation (Gillam et al., 2000). Thus, there are reasons to consider host and bacterial routes as two pathways of isatin biosynthesis.

Efficient functioning of the bacterial indole oxidizing machinery in the gut has been demonstrated using intra-cecal indole overload (500 mg/kg) (Jaglin et al., 2018). Administration of a large dose of indole to conventional Fischer 344 rats increased brain levels of isatin and caused behavioral changes typical of isatin-induced sedation (Jaglin et al., 2018). The increase of brain isatin from basically undetectable levels was comparable with the intraperitoneal administration of 50–100 mg/kg isatin (Jaglin et al., 2018). This convincingly indicates a significant role of gut microbiota in generation of isatin for regulation of the brain functioning.

However, more than 30 years ago Sandler et al. (1991) demonstrated that isatin levels in the brain, heart, liver, and kidney of germ free rats and conventional animals of the same strain (Lister hooded rats) insignificantly differed. Only the urinary level of isatin was more than fortyfold lower in germ free rats than in Lister hooded conventional rats. Besides evident interstrain differences (Fischer 344 vs. Lister hooded rats) this suggests that the host isatin story becomes more complicated because “normal” isatin production under germ free conditions strictly depends on exogenous (dietary?) indole resources. Such possibility cannot be ruled out because plants (e.g., maize) contain Trp synthase catalyzing a reaction of indole-3-glycerol phosphate cleavage to indole and glyceraldehyde-3-phosphate (Kriechbaumer et al., 2008). Otherwise, we can only hypothesize that some known host enzyme with flexible substrate specificity can perform the initial stage of tryptophan cleavage yielding free indole.

Considering decisive contribution of bacteria to the level of urinary isatin, some data indicate, that the level of isatin, produced by gut microbiome, may be pharmacologically regulated. At least, the effect of dexamethasone normalizing urinary isatin output elevated under stress conditions (Tozawa et al., 1998) may be attributed to the glucocorticoid action on gut flora. The thing is that dexamethasone upregulates cytochrome P450 2A6 (Onica et al., 2008), the most active cytochrome P450 isoform in indole conversion to isatin. This means that the host pathway of isatin synthesis is not inhibited and the changes in urinary isatin obviously reflect glucocorticoid modulation of the gut microbiome (Huang et al., 2015).

It should be noted that the role of particular representatives of the gut microbiome in isatin formation in norm and pathology remains unknown. In an attempt to evaluate distribution of the tryptophanase gene, Jaglin et al. (2018) analyzed an integrated catalog of reference genes in the human gut microbiota. Although they found 373 *tna*A-like genes, more than 60% originated from unknown species and most of the assigned sequences derived from *Bacteroidaceae, Rikenellaceae, Clostridiaceae, Lachnospiraceae, Enterobacteriaceae*, and *Rhodobacteraceae* families (Jaglin et al., 2018). However, among these families only the *Enterococcaceae* family was found to be increased in the intestinal microbiota population of patients with Parkinson’s disease (Huang et al., 2021). Thus, further studies are needed to determine particular bacteria, responsible for (altered) isatin production.

## Conclusion

Isatin (indole-2, 3-dione), a tryptophan-derived signaling molecule, can be formed *via* the host and bacterial pathways. Experiments with radiolabeled [2-^14^C]indole, orally administered to rats, demonstrated that a significant proportion of the radiolabel (about 6%) was detected in urinary isatin (King et al., 1966). Taking into consideration that urinary isatin is mainly produced by gut microbiota (Sandler et al., 1991) and the intra-cecal indole overload is accompanied by the isatin increase in the brain (Jaglin et al., 2018), it appears that the bacterial pathway plays the major role in isatin biosynthesis. The pharmacological control of the urinary isatin level (Tozawa et al., 1998, 1999) consistent with known regulation of gut microbiome by glucocorticoids, benzodiazepines, and other pharmacological agents (Huang et al., 2015; Walsh et al., 2018) raises an interesting possibility on the isatin-mediated regulation of brain functions also *via* gut microbiome remodeling. However, one should take into consideration that the level of oxindole, another metabolite formed during indole oxidation (King et al., 1966; Jaglin et al., 2018; Figure 2), significantly exceeds the isatin level (Jaglin et al., 2018). In this context, the effects of oxindole and isatin on gut microbiome should be reliably distinguished.

There is increasing evidence that isatin is a multifunctional agent that can potentially act on numerous targets. Besides individual biological targets, demonstrating the highest sensitivity to physiologically achievable concentrations of isatin (natriuretic peptide receptors (NPR) and NPR-coupled guanylate cyclase, NO-stimulated guanylate cyclase, monoamine oxidase B) (see for review Medvedev et al., 2018), there is a group of receptors exhibiting rather weak sensitivity to isatin. Such weak inhibition of multiple targets may contribute to some behavioral effects of isatin reported in the literature.

Considering isatin as a multifunctional agent, it should be noted that isatin influences various protein-protein interactions (PPI). In the absence of isatin, isatin-binding proteins mainly exist as independent molecules unrelated to each other. Recent bioinformatics analysis of proteomic profiling data has shown that after administration of isatin most brain isatin-binding proteins formed clusters that were not detected in the brain before administration of this signaling molecule (Medvedev et al., 2020). It is particularly interesting that isatin promoted PPIs between protein partners, each of which did not interact with isatin (Ershov et al., 2017, 2020). Ternary complex formation, in which isatin linked ferrochelatase (FECH) and adrenodoxine reductase (ADR), FECH/isatin/ADR, was characterized by higher affinity as compared with complex formation observed without isatin. This is a new regulatory mechanism, by which isatin can modulate PPI in the brain. It may be considered as a novel molecular basis for changes in behavioral reactions induced by increased isatin concentrations.

## Author Contributions

AM and OB: data analysis, original draft preparation, review, and editing. Both authors contributed to the article and approved the submitted version.

## Conflict of Interest

The authors declare that the research was conducted in the absence of any commercial or financial relationships that could be construed as a potential conflict of interest.

## Publisher’s Note

All claims expressed in this article are solely those of the authors and do not necessarily represent those of their affiliated organizations, or those of the publisher, the editors and the reviewers. Any product that may be evaluated in this article, or claim that may be made by its manufacturer, is not guaranteed or endorsed by the publisher.
